# A Tale of Two Transcriptomic Responses in Agricultural Pests via Host Defenses and Viral Replication

**DOI:** 10.3390/ijms22073568

**Published:** 2021-03-30

**Authors:** Pramod Pantha, Subbaiah Chalivendra, Dong-Ha Oh, Bret D. Elderd, Maheshi Dassanayake

**Affiliations:** Department of Biological Sciences, Louisiana State University, Baton Rouge, LA 70803, USA; ppanth1@lsu.edu (P.P.); subbaiahchalivendra@gmail.com (S.C.); ohdongha@lsu.edu (D.-H.O.)

**Keywords:** baculovirus, hemolymph, chitin metabolism, extracellular matrix organization, cuticle development, biopesticides

## Abstract

Autographa californica Multiple Nucleopolyhedrovirus (AcMNPV) is a baculovirus that causes systemic infections in many arthropod pests. The specific molecular processes underlying the biocidal activity of AcMNPV on its insect hosts are largely unknown. We describe the transcriptional responses in two major pests, *Spodoptera frugiperda* (fall armyworm) and *Trichoplusia ni* (cabbage looper), to determine the host–pathogen responses during systemic infection, concurrently with the viral response to the host. We assembled species-specific transcriptomes of the hemolymph to identify host transcriptional responses during systemic infection and assessed the viral transcript abundance in infected hemolymph from both species. We found transcriptional suppression of chitin metabolism and tracheal development in infected hosts. Synergistic transcriptional support was observed to suggest suppression of immune responses and induction of oxidative stress indicating disease progression in the host. The entire AcMNPV core genome was expressed in the infected host hemolymph with a proportional high abundance detected for viral transcripts associated with replication, structure, and movement. Interestingly, several of the host genes that were targeted by AcMNPV as revealed by our study are also targets of chemical insecticides currently used commercially to control arthropod pests. Our results reveal an extensive overlap between biological processes represented by transcriptional responses in both hosts, as well as convergence on highly abundant viral genes expressed in the two hosts, providing an overview of the host–pathogen transcriptomic landscape during systemic infection.

## 1. Introduction

Baculoviruses are highly virulent arthropod-specific viruses [1]. They usually have specific host ranges and most of them only infect congeneric insect species. Autographa californica Multicapsid Nucleopolyhedrovirus (AcMNPV) is the most notable exception in the Baculoviridae family of viruses, which infects over 35 species belonging to 11 lepidopteran families [2].

Baculoviruses have large rod-shaped nucleocapsids with circular DNA genomes. The sequenced AcMNPV genomes are ~134 kbp in size and contain ~150 tightly spaced genes [3,4]. AcMNPV is widely used as a molecular tool in gene therapy; a vector in engineered protein production using insect cell cultures; and a potent biopesticide in integrated pest management systems that could spare beneficial insects, especially in ecologically sensitive areas [5].

AcMNPV is found as two distinct virion phenotypes [6]. First, the occlusion-derived virus is transmitted among insects primarily via horizontal transmission when uninfected hosts inadvertently consume the virus. If enough virus is consumed, a fatal infection occurs. The virus replicates within the larva until the virus triggers the liquefaction of the insect host, which releases occlusion-derived viruses onto nearby foliage [7]. After the virus is released to the environment, uninfected larvae eat the newly contaminated foliage and the cycle continues [8]. Second, within the insect host following infection of the midgut epithelial cells, the budded form of the virus causes secondary infection in the open circulatory system and, subsequently, invades cells in other tissue types [9]. Once an individual larva is infected, the larva does not continue to grow or molt to larger instars, whereas uninfected individuals do. Besides horizontal transmission, vertical transmission between mother and offspring may also occur. However, vertical transmission often results in a “covert” infection that does not kill the host [8].

The fall armyworm (*Spodoptera frugiperda*) and the cabbage looper (*Trichoplusia ni*) are among major agricultural pests vulnerable to AcMNPV infection. These two pests together pose a significant threat to global food security, affecting over 150 crops including corn, rice, soybean, and cotton. The total yield loss by *S. frugiperda* alone in 12 maize producing African countries in 2017 was estimated to be between US$2.48 and $6.19 billion [10]. If appropriate control measures are not applied, these pests together can exacerbate the problem of food security and livelihood of many small farmers worldwide due to their wide host range. They are difficult to control due to their rapid spread and the development of their resistance to many insecticides [11,12]. Therefore, AcMNPV strains that can naturally infect these serious agricultural pests offer a promising mode of pest control. However, it is imperative to understand the mode of infection, disease progression, and epidemiology of a naturally occurring virus before its commercialization, to minimize unintentional secondary effects [12].

Both host species are widespread multivoltine (i.e., multiple generations per year) pests from the same family Noctuidae [13,14,15]. After hatching, *S. frugiperda* has six larval instars or development stages before the larvae pupate and later emerge as adults, whereas *T. ni* has five larval instars [16,17]. These two pests are readily infected in nature by baculoviruses, particularly when they reach large population densities [1].

In vivo studies investigating the genetic basis for AcMNPV infection and the integrated host responses are quite limited. Due to difficulties in generating the synchronized samples post infection, there are very few studies on the systemic infection phase of AcMNPV on host and viral transcription. Most studies exploring transcriptional regulation of these host–pathogen interactions use cell cultures infected with the virus. The transcriptome responses of *S. frugiperda* [18,19] and *T. ni* [20,21] cell cultures infected with AcMNPV have shown quite divergent transcriptional profiles, which makes it difficult to deduce the impact of these responses in intact organisms. Recently, Shrestha et al. described the in vivo transcriptional response of *T. ni* during AcMNPV infection [22]. They reported the oral to midgut tissue-specific transcriptomic responses at the primary stage of infection in 5th instar larvae. In vivo studies that explore the transcriptional dynamics in response to AcMNPV infections appear to be even fewer in *S. frugiperda*. To our knowledge, studies exploring gene expression profiles of systemic AcMNPV infections in intact hosts along with reciprocal viral transcriptomes are absent either in *S. frugiperda* or *T. ni*.

In this study, we provide reference transcriptomes for the hemolymph in 4th instar larvae and report the host–pathogen transcriptional responses associated with the systemic infection phase as represented in the hemolymph in *S. frugiperda* and *T. ni* during AcMNPV infection. The transcriptional profiles in the host hemolymph captured in our data link host responses to the virus as well as the reciprocal viral responses to the hosts. Our results indicate major transcriptional changes to support initiation of critical cellular and developmental adjustments in the hosts during pathogenesis.

## 2. Results

### 2.1. Lethal Effects of AcMNPV on S. frugiperda and T. ni

**Figure 1:**
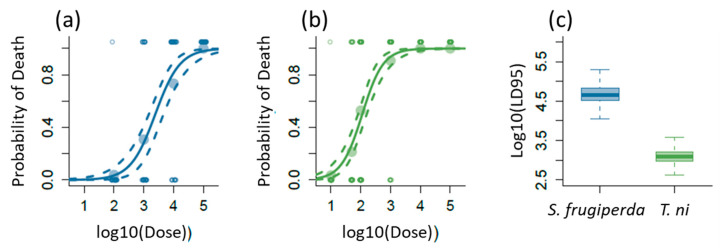
Lethal AcMNPV dose determination for *Spodoptera frugiperda* and *Trichoplusia ni*. The effects of increasing doses of baculoviruses on the probability of larval death for (**a**) *S. frugiperda* and (**b**) *T. ni* along with the corresponding (**c**) box plot of the lethal dose at which 95% of the individuals would be expected to die identified as LD95. For (**a**) and (**b**), the solid line is the median dose-response curve and the dashed lines are the 95% credible intervals for the curve. The large filled points represent the mean response for each dose and the small open points are the individual data. These data are jittered for ease of presentation. For [c] the dark line of the box plot is the median with the box encompassing the interquartile range between the first and third quartiles and the whiskers represent 1.5 times the interquartile range.

Clearly, both *S. frugiperda* and *T. ni* were adversely affected by increased doses of AcMNPV (Figure 1a,b), resulting in the death of a large proportion of larvae at higher doses. Despite these two insects being from the noctuidae family, *S. frugiperda* required a much larger dose of the virus to become infected as compared to *T. ni* (Figure 1a,b). Additionally, *S. frugiperda* has an LD50 (lethal dose at which 50% of the larvae would be expected to succumb to viral infection) of 3.4 and *T. ni* has an LD50 of 2.0, which is similar in magnitude difference to the LD95 (lethal dose at which 95% of the larvae would be expected to succumb to viral infection). Given the relatively good fit of the logistic model to the data and the relatively narrow credible intervals, the median LD95 for both species was reasonably well estimated. For the subsequent transcriptome analysis, we infected *S. frugiperda* and *T. ni* using the estimated species-specific LD95, 10^4.5^ and 10^3^ occlusion bodies, respectively, using the same diet cube method as used in the dose-response experiment (see the Materials and Methods section). Note that there is over a 10-fold difference in the LD95 between the species (Figure 1c).

### 2.2. De Novo Assembly and Annotation of S. frugiperda and T. ni 4th Instar Reference Transcriptomes

We report the most curated reference transcriptomes that represent the hemolymph tissue of *S. frugiperda* and *T. ni* currently available. On average, 62 million raw reads were obtained for each RNA-seq sample generated for *S. frugiperda* and *T. ni* (Supplementary Table S1). The fully assembled transcriptomes are available at NCBI BioProject PRJNA664633. We selected 17,908 *S. frugiperda* transcripts (mean length 1458 nt) and 19,472 *T. ni* transcripts (mean length 1773 nt) to represent the protein-coding reference transcriptomes (Table 1). The number and length distribution of total protein-coding transcript models in the current reference transcriptomes (Supplementary Figure S1a,b) were comparable to the protein-coding transcripts available for *Bombyx mori* [23], *Helicoverpa armigera* [24], *Spodoptera litura* [25], and the genome of *T. ni* [26] (Supplementary Figure S1c,d). Our predicted protein coding transcripts mainly contained complete open reading frames (ORFs) with start and end codons included in the transcript model (Supplementary Figure S2a). We were able to map >75% of the initial RNA-seq reads to the reference transcriptomes for both species (Supplementary Table S2).

As *D. melanogaster* genes provided the highest number of functional attributes available for an arthropod model, we first annotated 5878 *S. frugiperda* and 6219 *T. ni* transcript models based on the *D. melanogaster* reference models when possible (see methods). The NCBI insect-Refseq database was used to annotate another 9273 transcripts from *S. frugiperda* and 9751 transcripts from *T. ni* (Supplementary Figure S3a). The remaining transcripts were subjected to BLASTX [27] against the NCBI-nr databases to annotate 1278 *S. frugiperda* and 888 *T. ni* transcripts. A final pool of remaining transcripts that did not show convincing similarity to other known eukaryotic transcripts (1479 *S. frugiperda* and 2614 *T. ni* transcripts) were annotated as “unknown putative proteins.” The annotation of reference transcriptome for both species is provided in the Supplementary Table S3.

We assessed the completeness of the reference transcriptomes based on the expected presence of core genes in metazoans as identified by the BUSCO database [28]. *S. frugiperda* and *T. ni* reference transcriptomes were found to have 80.6% and 82.1% expected BUSCOs, respectively, suggesting that these transcriptomes contain a core gene component comparable to those in the high quality lepidopteran genome available for silkworm [29] (Supplementary Figure S2b). Furthermore, our *S. frugiperda* reference transcriptome showed a better BUSCO representation than the previously published *S. frugiperda* genome and transcriptome assemblies [30,31] (Supplementary Figure S2b). Only 36% of RNA-seq reads generated for *T. ni* in our study mapped to a genome assembly recently made available for this insect [26], compared to the 84% of mapped reads to our reference transcriptome. These comparisons confirm the appropriateness of the use of our reference transcriptomes for our downstream analyses.

### 2.3. Host Transcriptomic Responses to the AcMNPV Infection

**Figure 2:**
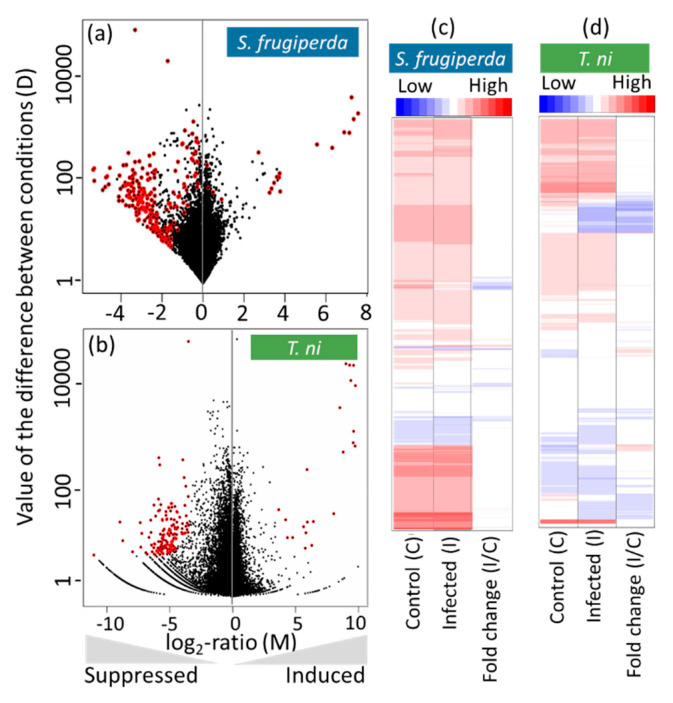
Host transcriptomic response to AcMNPV infection. Summary MD plots of the normalized expression values for control and AcMNPV infected samples for (**a**) *S. frugiperda* coding transcripts (18 up- and 157 down-regulated transcripts) and (**b**) *T. ni* coding transcripts (20 up- and 118 down-regulated transcripts). Differently expressed transcripts (DETs) at a q-value cutoff of 0.95 are indicated in red dots and black dots, which represent non-significant transcript expression changes. All DETs with their respective fold changes are listed in the Supplementary Table S4. Heatmaps show log_2_ normalized expression of (**c**) 17,873 *S. frugiperda* and (**d**) 18,203 *T. ni* protein-coding transcripts in control and infected samples followed by fold changes in the third column. The genes are clustered based on their expression strength similarity indicated in the scale from low (blue) to high (red) normalized expression.

We identified 175 *S. frugiperda* differently expressed transcripts (DETs) and 138 *T. ni* DETs in response to the AcMNPV infection in hemolymph samples (Figure 2a,b). Our results represent the hemolymph samples pooled from 30 individuals 30 hours post feeding with relevant diet cubes per treatment/sample (see Methods). The DETs represent ~1% *S. frugiperda* and ~0.7% *T. ni* of respective reference transcriptomes. The relatively small sets of differently co-expressed genes suggest that the observed transcriptomic response pertains to an active host responding to the infection, rather than largely misregulated transcriptomes represented in a dead or a dying host overrun by the pathogen. In addition, the transcriptional responses between control and infected larvae within a host species differed minimally relative to the differences between basal transcriptomes of the two hosts (Supplementary Figure S4). The wide divergence observed in the basal transcriptomes of the two host species is not surprising, since they belong to two different genera. Our aim was to identify the common response mechanisms or pathways deduced from DETs independently detected in the two host species to the viral pathogen. In both host species, more transcripts were suppressed compared to those that were induced by viral infection (Figure 2). This is consistent with the trend observed in previous studies that investigated transcriptomic responses of *S. frugiperda* and *T. ni* infected with AcMNPV in cell cultures, other tissues, or other developmental stages [18,19,20,21,22].

Overall, 83.4% *S. frugiperda* and 89.1% *T. ni* DETs could be assigned to functionally informative annotations. This was based on either functional validation of a putative homolog in *D. melanogaster* or a homolog reported with a putative function in another lepidopteran host. The number of Gene Ontology (GO) annotations were used when available but was more limited as GO annotations largely depended on the sequence similarity of *S. frugiperda* and *T. ni* transcript models to a *D. melanogaster* gene that also had an assigned GO term. In the following sections, we highlight the shared host transcriptomic responses via enriched functional processes based on clustering of functional annotations of DETs. All DETs with functional annotations that had a fold change of 4 or more in response to the AcMNPV infection were considered. The full list of DETs and their assigned GO terms (when available) are presented in Supplementary Table S5.

### 2.4. Transcripts Associated with Chitin Metabolism and Epithelial Membrane Formation and Stability Were Suppressed in AcMNPV-Infected Hosts

**Figure 3:**
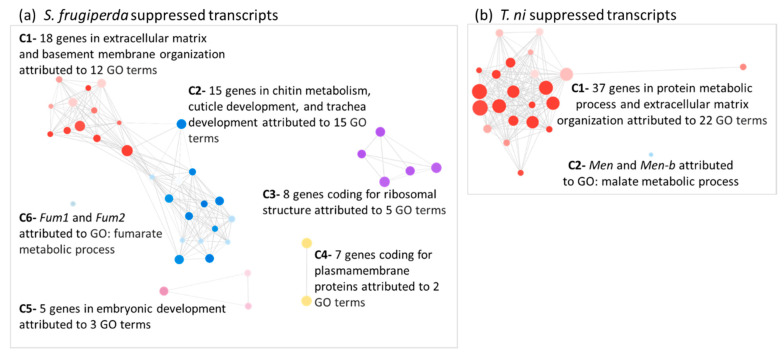
Overview of enriched functional processes represented by suppressed genes in the infected host transcriptomes. Functional clusters of (**a**) *S. frugiperda* and (**b**) *T. ni* transcripts suppressed upon AcMNPV infection. Each network shows GO terms, marked as nodes connected by edges that represent a minimum overlap of 80% genes (in the smaller GO term of the pair) based on Markov clustering (MCL). Distinct colors indicate shared functional groups within the network. The radius of the node represents the number of genes and the shade represents FDR adjusted *p*-value of <0.05 enrichment assigned using GOMCL [41]. Each cluster is named based on the largest enriched GO term in a given cluster.

**Figure 4:**
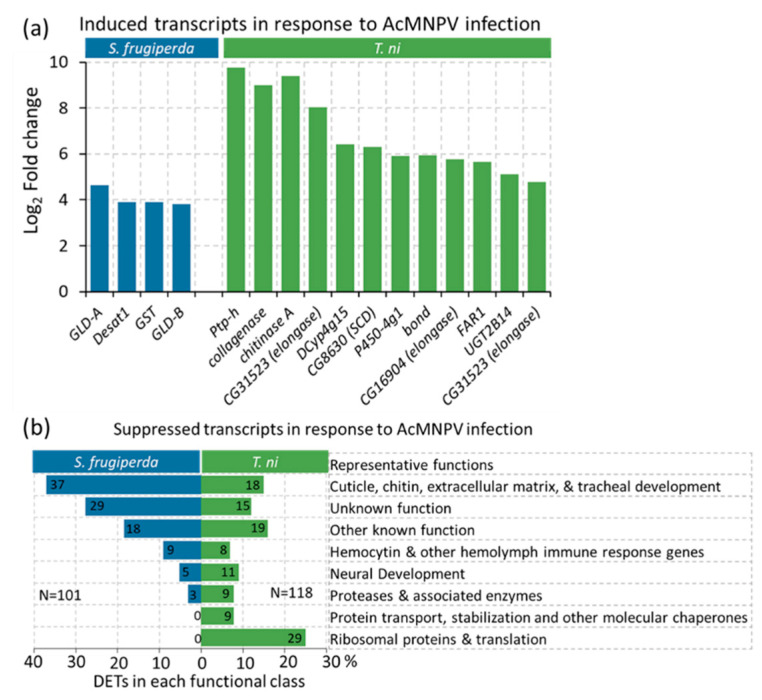
*S. frugiperda* and *T. ni* differently expressed transcripts (DETs) in the hemolymph in response to the AcMNPV infection. (**a**) Induced DETs and (**b**) suppressed DETs summarized based on functions to represent a total of 101 in *S. frugiperda* and 118 in *T. ni* transcripts. Log2 fold changes are calculated based on control groups within species.

The two largest enriched functional clusters out of six in *S. frugiperda* and the largest cluster of the two in *T. ni*, represented in the “suppressed” set of genes reveal a coordinated down-regulation of chitin-related genes (Figure 3). Chitin metabolism and its associated pathways are central to the formation and stability of the peritrophic matrix, cuticle, and the tracheal system that are in contact with the hemolymph [32]. The down-regulated genes that are associated with chitin include *chitin synthase 1* (*Chs1/kkv*); genes encoding chitin-binding proteins especially in the peritrophic matrix (*Gasp*) and other genes known for their chitin associated functional roles in cuticle development such as the *Osiris* gene family members [33,34,35,36] (Figure 4b and Supplementary Table S4). Interrupted chitin metabolism at the cellular level is tightly coupled to the organ integrity. *Drosophila chs1* mutants with suppressed expression show defective tubular structure, irregular tracheal epithelial tube expansion, and irregular subapical cytoskeletal organization [37]. The host genes *serpentine* (*serp*) and *vermiform* (*verm*) that bind to chitin and modify its surface play significant roles in the tracheal tube development [38,39,40] together with *uninflatable* (*uif*), which regulates tracheal growth and molting. These and many other cuticle- and tracheal growth-related genes were highly co-suppressed in infected host tissue (Figure 4b and Supplementary Table S4).

The budded viruses exiting the midgut epithelial cells need to penetrate the basement membrane of the gut epithelium before entering the hemocoel and then the basement membrane of tracheal cells for systemic infections [40]. Therefore, genes associated with collagen metabolism and other integral components of the basement assembly are expected candidates for virus regulated transcriptional processes in the host. Transcripts coding for structural components of the extracellular matrix including collagen were among the most significantly suppressed in response to the AcMNPV infection in both species (Figure 4b and Supplementary Table S4). We observed multiple transcripts associated with glycoproteins, likely formed in hemocytes that function in basement membrane stability, highly suppressed coordinately in both species during AcMNPV infection. Among them, *laminins*, *osteonectins* (*SPARC*), and *papilins* are notable. Laminin is the most prevalent glycoprotein in the basement membrane and is also found in extracellular matrices of tracheal cells. It is formed of three chains coded by *LanA*, *LanB1,* and *LanB2* [42,43]. Notably, we found transcripts that represent all three *Laminin* chains to be coordinately down-regulated in the infected tissue in both species (Supplementary Table S4). *SPARC,* known as a Ca^2+^-binding extracellular glycoprotein that modulates cellular interactions with the extracellular matrix, was also down-regulated in both hosts (Supplementary Table S4). *SPARC* is particularly expressed during cellular injury or wounding that require tissue remodeling and functions in basal lamina assembly and stability [44,45]. Similarly, transcripts potentially coding for papilins were co-suppressed in infected samples of both hosts (Supplementary Table S4). The coordinated suppression of chitin and basement membrane associated glycoproteins in our results suggest a transcriptomic signal for weakened membrane stability in infected host tissue during the systemic infection of AcMNPV.

### 2.5. Transcripts Associated with Hemocyte-Induced Defenses and Immune Responses Were Suppressed during Systemic Infection

*Hemocytins* and *hemolectins* were among the most highly suppressed genes in both *S. frugiperda* and *T. ni* infected samples (Figure 4b and Supplementary Table S4). Membrane damage in contact with the hemocoel is sensed by hemocytes and these can initiate immune responses during pathogen invasions. *Hemocytin* is a key gene that mediates hemocyte aggregation and hemolymph melanization in lepidopteran innate immunity against pathogens [46,47,48]. Similarly, *Hemolectin* is specifically expressed in larval hemocytes, acts as a clotting factor, and is known to initiate immunity responses during pathogen infections [49,50]. Additionally, hemolymph proteases are known for their pivotal roles in defense responses against many pathogens as well as in development processes such as molting [51]. The specific regulatory pathways of many of these proteins are not definitive yet, but their collective role as a functional group in insect immunity and development are established. We found multiple proteases in both infected hosts highly suppressed as a prominent group among all suppressed transcripts (Figure 4b and Supplementary Table S4).

### 2.6. Lipid Metabolism and Oxidative Stress Responses Emerge as the Most Prominent Functional Processes Induced by Both Hosts in Response to Infection

The lipid biosynthesis pathways not only affect lipid membranes, but also many other primary biological processes related to energy metabolism and signaling pathways. Interestingly, *Desaturase1 (Desat1*), reportedly tightly regulated at the transcriptional level [52] and known to be required for the biosynthesis of unsaturated fatty acids, is induced in *S. frugiperda*. Several other fatty acid modification enzymes, e.g., elongases such as *jamesbond/bond* and *CYP4G*, are co-induced in *T. ni* (Figure 4a). It is notable that *bond* and *CG16904* together were assigned to 60 GO-terms, exemplifying their influence in multiple biological functions linked to their primary molecular functions in lipid metabolism (Supplementary Figure S5 and Supplementary Table S5).

Reactive oxygen species (ROS) generation and induction of oxidative stress are inevitable when host membranes are disrupted during host–pathogen interactions. Supportive of this expectation, all three genes induced in the infected *S. frugiperda* hemolymph in addition to *Desat1* (i.e., above a four-fold expression change) relate to oxidative stress (Figure 4a). These include transcripts coding for a cytosolic *GST* and two *FAD-glucose dehydrogenases (GLD).* GSTs form a broad family of critical defense proteins against oxidative stress [53,54] and *GLD* can induce ROS generation as a defense response. Aligned with our observations, a recent study showed that *GLD* is induced as a defense response during AcMNPV infections in *Helicoverpa zea* [55].

### 2.7. AcMNPV Genome Response to the Insect Hosts

To check whether viral sequences were present in our hemolymph samples, we mapped RNA-seq reads from both species to the published AcMNPV genome [4] (Supplementary Table S2b). As expected, viral sequences were detected almost exclusively in the infected samples. We mapped 1.13% and 7.41% of total reads from infected *S. frugiperda* and *T. ni* samples, respectively, to the AcMNPV genome. It was interesting that a small number of reads from *T. ni* control samples (<0.01%) were mapped to the AcMNPV genome (Supplementary Table S2b). While it is not conclusive that these could represent domesticated viral genes expressed at low levels in the *T. ni* genome, previous studies have indicated that AcMNPV genes are found in arthropod genomes as a result of horizontal gene transfer [56,57]. The difference in the percentage of mapped reads to the AcMNPV genome between two hosts is likely due to the host-specific differences. We infected both larvae at their 4th instar stage for the same amount of time before taking the hemolymph samples. Furthermore, we ranked the abundance of AcMNPV genes in both infected hosts (Supplementary Table S6). Interestingly, many of these AcMNPV genes showed similar ranks in their relative expression abundances despite being expressed in two different hosts, implying similarities in how the viral infection may be progressing in the hemolymph (Supplementary Table S6).

**Figure 5:**
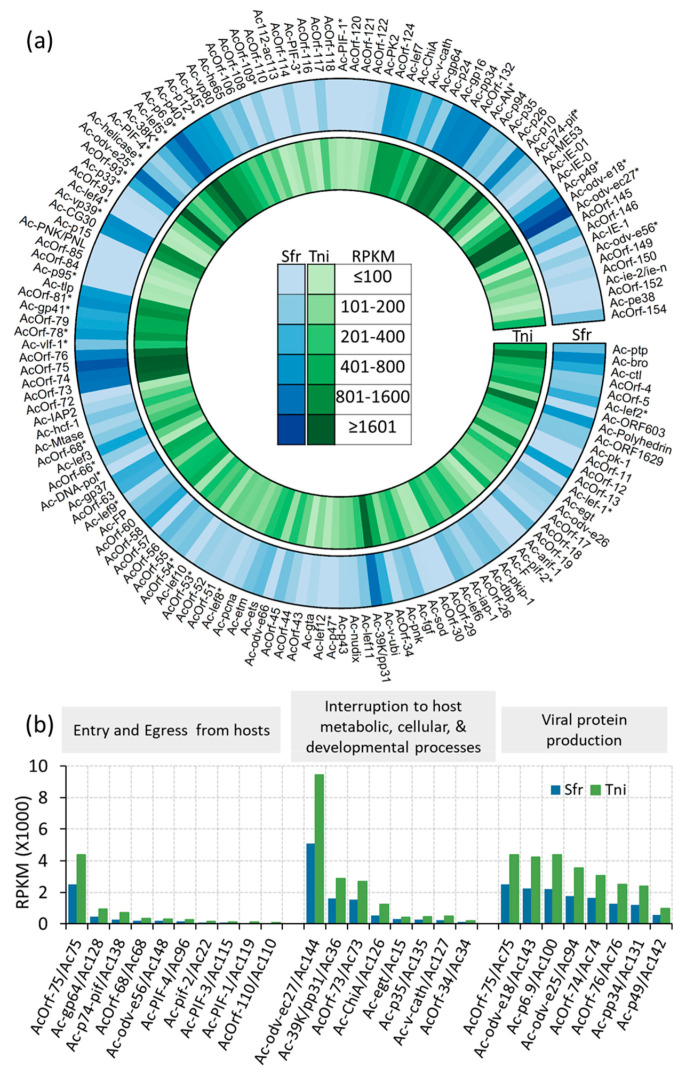
The AcMNPV genome expressed in the host hemolymph. (**a**) The circular plot shows the normalized gene expression of AcMNPV genes in infected *S. frugiperda* (Sfr) and *T. ni* (Tni) infected hosts. Core baculovirus genes are marked with asterisks. (**b**) Expression of AcMNPV genes associated with entry and egress from insect hosts, interruption to host metabolic, cellular, and developmental processes, and viral structural proteins.

The AcMNPV strain E2 genome has 149 protein-coding genes [4]. We detected 148 genes in our viral transcriptome expressed in the hemolymph and *Ac-IE-1/Ac147* was not expressed in both species (Figure 5a and Supplementary Table S6). Although we could not detect *Ac-IE-1/Ac147* in our transcriptome data, we found both *Ac-IE-0* and *Ac-IE-01* were expressed (Figure 5a and Supplementary Table S6) that were shown to have similar functions to *Ac-IE-1* in previous studies to produce the infectious virions [58,59]. Notably, *Ac-IE-1/Ac147* and *Ac-IE-0* share a large part of the reading frame [60] (Supplementary Figure S6). The expression of *Ac-IE-1* and *Ac-IE-0* were shown to be induced during early phase of the infection in *Sf9* cell lines [61]. Transcripts associated with the budded virus compared to the occlusion-derived virus are reportedly more prominent during the systemic infection phase [6]. In agreement with this previous finding, we observed higher expression of viral genes related to the formation of the budded virus than those involved in the production of occlusion-derived virus (Supplementary Table S6).

In the infected hemolymph tissue, we found viral genes that mark both early and late stages in their expression sequence. For example, a chromatin-like structure called the virogenic stroma is formed in the center of the nucleus of infected cells. *Ac36/Ac-39K/pp31* is an early viral gene reported to be among the two primary viral genes that initiates this morphological change in the host cells [62,63]. In contrast *Ac74/AcOrf-74/Bm60,* required for the budded virus production and also found in nucleocapsids of both budded and occlusion-derived virions, is thought to be expressed at a late stage [64]. Both *Ac36/Ac-39K/pp31* and *Ac74/AcOrf-74* are among the top 10 highly expressed viral genes in infected samples of both hosts (Supplementary Table S6). The baculovirus genes are sometimes reported to be expressed as multi transcript units, but the related mechanisms or their defined borders for transcriptional units are poorly understood [20]. The majority of the highly abundant viral transcripts in our study were associated with the production of nucleocapsid and envelope proteins. Many such integral proteins of the nucleocapsid or envelope are known to function in viral entry and exit pathways (Figure 5a and Supplementary Table S6).

The viral genes affect cellular, metabolic, and developmental alterations in the host in addition to initiating viral replication and virion movement in the host cells. Some of these virus-induced host metabolic processes include host membrane degradation and developmental arrest that stops molting. The co-expressed viral genes *chitinase* (*Ac126/Ac-ChiA*) and *cathepsin* (*Ac127/Ac-v-cath*) are required for the liquefaction of hosts; chitinases degrade host chitins and cathepsins serve as broad-spectrum proteases that degrade host tissue [65]. Both *Ac126/Ac-ChiA* (found at RPKM of 540 in *S. frugiperda* and 1227 in *T. ni*) and *Ac127/Ac-v-cath* (found at RPKM of 236 in *S. frugiperda* and 494 in *T. ni*) were highly expressed in the infected samples in our study (Figure 5b and Supplementary Table S6). This indicates a strong transcriptional signal about the extensive tissue damage initiated in the host by the virus along with the reciprocal transcriptomic signals in the hosts that suggest interrupted membrane stability during systemic infection.

*Ac144/Ac-odv-ec27* is the most highly expressed AcMNPV gene (expressed at RPKM of 5081.6 in *S. frugiperda* and 9456.6 in *T. ni*) found in infected hosts in our study (Figure 5a). This is a key gene encoding a major virion structural element [66].

### 2.8. Viral-Host Co-Transcriptional Interactions

Several AcMNPV transcripts and their associated proteins are known to directly interact with host proteins to regulate pathogenicity. We wanted to assess whether such host–parasite transcript interactions could be elucidated from comparing viral transcripts co-expressed with host transcripts in the infected hemolymph. Host lipids play multifarious roles in a virus life cycle, right from the entry of the virus into host cells by endocytosis, during replication in protected membrane vesicles, and until the virions exit the cell by exocytosis. For example, host fatty acid desaturases are required for virus replication to alter the fluidity and plasticity of membranes for viral replication complexes [67]. As described earlier, host *Desat1*, along with several transcripts associated with fatty acid synthesis, are up-regulated in the infected hosts.

Viral entry and egress pathways highly depend on cell shape, entry and exit to the nucleus, and microvesicles regulated by host actins [57,68]. A late viral gene, *Ac34/AcOrf-34* induces nuclear actin polymerization that promotes virus replication, and nuclear export of the virus [69,70,71]. In our study, *Ac34/AcOrf-34* is a highly abundant viral transcript present in the hemolymph. Reciprocally, we observed a marginal induction in *S. frugiperda Act57B* (Supplementary Table S4). *Act57B* is a major myofibrillar actin gene expressed during larval stages in *Drosophila* and encodes a major structural protein found in the hemolymph [72]. Previous studies have reported that *Ac34/AcOrf-34* directly regulates the host actin-associated Arp2/3 protein complex in the nucleus [69]. We detected a 100-fold suppression in the levels of transcripts expected to code for the Arp2/3 complex in infected *T. ni* hosts (Supplementary Table S4). Expression of a couple of transcripts coding for zipper and cytoplasmic myosin light chain proteins, also known for their roles in regulating cell shape, was reduced by over 1800-fold in the infected *T. ni* hemolymph (Supplementary Table S4).

Apoptosis is an important cellular process induced as a defense mechanism against viral infections by insect hosts [73]. *Hcf-1/Ac70, Ac-p35/Ac135, Ac-iap-1/A27*, and *Ac-IAP2/Ac71* AcMNPV genes were previously described for their role in determining host tolerance to infections [73,74,75,76]. Host-cell specific factor (*Hcf-1/Ac70* with normalized expression at 243.4 and 422.9 RPKM in *S. frugiperda* and *T. ni*) (Figure 5a and Supplementary Table S6) is known to be required for viral DNA replication [74]. Two cell lines of *T. ni* (TN-368 and BTI-TN5B1-4) in a previous study has shown different levels of infection severity governed by *hcf-1* [74]. The *T. ni* larvae infected with AcMNPV harboring a loss of function *hcp-1* mutant allele was more tolerant compared to the wild type allele. However, similar responses were not detected when SF-21 cell lines or *S. frugiperda* larvae were used as viral hosts [74]. We found *Ac-p35/Ac135* at similar expression levels to *hcf-1* in both species (270.8 and 469.3 RPKM in *S. frugiperda* and *T. ni*) (Figure 5a and Supplementary Table S6). Previous studies report that *p35* mutated AcMNPV required higher doses of virus for LD50 when it was used to infect *S. frugiperda,* but did not show a difference in virulence when used to infect *T. ni* [76]. The known inhibitors of apoptosis *Ac-iap-1/*A27 (expressed at 148.9 and 243.2 RPKM in *S. frugiperda* and *T. ni*) and *Ac-IAP2/*Ac71 (expressed at 229.3 and 407.2 RPKM in *S. frugiperda* and *T. ni*) also showed close to two-fold difference in relative abundance in the two hosts in our study (Figure 5a and Supplementary Table S6). *Ac-IAP-1* and *Ac-IAP-2* are known to inhibit apoptosis in Tn-Hi5 and *Sf9* cells [27,77]. However, *S. frugiperda* and *T. ni* larvae infected with *Ac-iap-1* loss of function AcMNPV mutant strain did not show a significant change in lethality compared to wild-type AcMNPV [78]. These may play important roles in regulating the AcMNPV infection strength in a host-specific manner in agreement with previous publications. We found reciprocal coordinated suppression of several host transcripts associated with apoptosis in infected hosts. For example, *calreticulin* (*Calr*)*, GDP dissociation inhibitor* (*Gdi*)*,* and *death-related protein* (*Drp*) were coordinately suppressed in infected *T. ni* hemolymph (Supplementary Table S4). Notably, the characteristic host apoptosis marker genes known for their defense were absent in the transcripts identified as significantly induced in the infected hosts. Therefore, we see a bias in the host transcriptomic signals towards an overall suppression of host apoptosis as a counter defense mechanism, favoring virus propagation (Supplementary Table S4).

AcMNPV-induced developmental arrest in the host is a known outcome in infected instars. In support of this expectation, we observed multiple host transcripts associated with larval developmental arrest. For example, the insect juvenile hormone synthesis genes, *adenosylhomocysteinase* and *farnesyl pyrophosphate synthase*, and transcripts encoding the heme peroxidase, *Cysu,* required during wing maturation, were co-suppressed in the infected *S. frugiperda* and *T. ni* hemolymph (Supplementary Table S4). Similarly, *Ac15/Ac-egt*, a highly abundant viral gene in infected hosts (RPKM of 295.8 in *S. frugiperda* and 433.1 in *T. ni*, Figure 5b and Supplementary Table S6) codes for the EGT enzyme that inactivates the insect molting hormone, ecdysone, that would lead to host developmental arrest [79].

## 3. Discussion

### 3.1. Host Transcriptomic Signatures Suggest Impaired Membrane Integrity, Enabling Viral Proliferation

**Figure 6:**
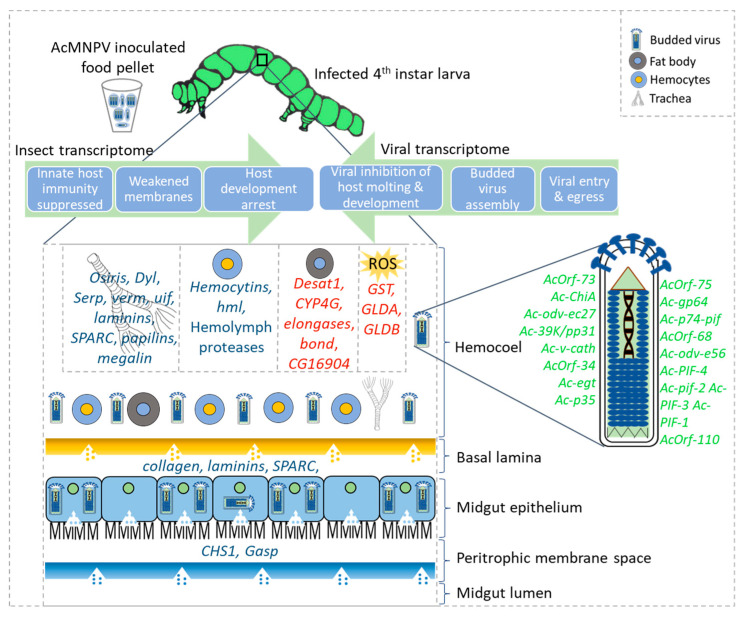
Overview of host and viral transcriptome responses in the hemolymph from 4th instar larvae of *S. frugiperda* and *T. ni* infected with AcMNPV. Prominent host genes that respond to the viral infection are listed in the cells/tissues most likely to express those genes. Induced genes are in red and repressed genes are in blue. Highly abundant viral genes are given in green.

Figure 6 provides an overview of genes and pathways affected by–viral interactions in the hemolymph at the systemic infection stage based on the collective deduction of our transcriptome-based analyses. Our results provide a compelling set of transcriptomic signals to support suppression of chitin-associated processes in the infected hosts, which can be linked to weakened membrane stability, as well as disrupted tracheal development during the systemic infection phase (Figure 3, Figure 4, and Figure 6). Chitin-centric processes are fundamental to the transcriptional regulation, which plays a key role in integrating various metabolic processes operating at the cell, organ, and organism levels during pathogenesis. The midgut epithelium tissue lining the hemolymph in contact with the tracheal system form the focal point for systemic infections by the budded virus [6,80,81]. The midgut epithelium and the tracheal system are lined by chitin and these tissues are both in contact with the hemolymph. The budded virus transiting the midgut epithelium propagate via the hemolymph and the tracheal system to cause systemic infections reaching all cells in the insect host [6,81,82]. The tracheoblasts, which are the smallest units in the tracheal system, are found in the hemolymph and also known to pierce the midgut basal lamina to oxygenate midgut epithelial cells. These are considered to be sites of AcMNPV secondary infection. Therefore, the midgut epithelium, basal lamina, chitin, the tracheal system, and the hemolymph play vital roles in establishing systemic infections [83,84,85]. Therefore, analyzing the transcriptional profile associated with chitin metabolism during host–pathogen interactions as suggested by [82] would be an important step in strategizing the development of baculovirus-based insecticides.

Chitinases are chitin-degrading enzymes often produced by both hosts and pathogens. Insect chitinases are required for organ morphogenesis, cell division, and development [80,82]. In our current study, we did not see a significant suppression of host chitinases, except for the induction of one transcript coding for a putative chitinase in *T. ni* (Figure 4a). The AcMNPV genome also codes for a chitinase that disrupt the cuticle and peritrophic matrix of the insect host [86]. Chitinases coded by baculovirus genomes have a greater sequence similarity to bacterial chitinases involved in fungal chitin degradation and are distinct from insect chitinases both in sequence as well as localization in host tissues [65,87]. A functional viral chitinase is critical to complete the infection cycle of the AcMNPV [88]. In our study, the AcMNPV chitinase gene, *Ac126/Ac-ChiA*, is highly expressed in both infected hosts (Figure 5a,b, and Supplementary Table S6). It is possible that the viral chitinase transcripts, together with the *cathepsin/Ac127/Ac-v-cath* transcripts required for liquefaction of the host, are transcribed early on during budded virus production.

The stability of basement membranes in the host is critical in mounting a structural barrier against the movement of the virus and containing the infection. Transcripts associated with the major glycoproteins (collagen, laminin, osteonectin, and papilin), known to function in basement membrane stability, were all coordinately suppressed in both infected hosts in our study (Figure 3, Figure 4b, Figure 6, and Supplementary Table S4). Laminin and type IV collagen are the dominant glycoproteins in the basement membrane and form a stable scaffold for other glycoproteins to create a network that provides both structural and signaling support to adjacent tissues [89,90,91,92,93]. These glycoproteins are required for membrane assembly, and facilitate tissue remodeling after damage to the membrane. They are found in fat bodies, basal lamina in the basement membrane, and in the extracellular layer secreted by epithelial cells and tracheal cells [42,45]. The baculovirus-encoded fibroblast growth factor is known to target the laminins in the basal lamina of tracheal cells, making them more susceptible to virus movement, and thereby facilitating systemic infections [94]. Therefore, in a progressive infection, damage to the basement membrane is unavoidable. In agreement with previous studies on baculovirus infections, we observed the coordinated down-regulation of multiple transcripts coding for both stable and dynamic components of the basement membranes (>15% of DETs) indicative of weakened structural barriers in the host.

Massive reorganization of lipid membranes is expected as the virus escapes from midgut to the hemolymph or from the hemolymph to tracheoblasts [84,95]. A recent study by Li et al. demonstrated that fatty acid biosynthesis was reduced at early disease stages and led to the reduction of virions in *S. frugiperda* Sf9 cell cultures, possibly as a host defense response [96]. This supports the proposal that fatty acid synthesis is a key process that modulates viral infection levels in host cells [97]. In our study, we observed transcripts involved in fatty acid modifications strongly induced in both hosts in response to the AcMNPV infection (Figure 4a and Figure 6). While induced host transcripts were much fewer compared to the suppressed transcripts (Figure 2a,b), it is notable that *Desat1*, stearoyl CoA desaturase, *elongases* (*bond*, *CG31523*, *CG16904*)*,* and transcripts potentially coding for cytochrome P450 (*CYP4G1*) that collectively function in lipid biosynthesis were among the few and most induced transcripts in the infected hosts (Figure 4a). Taken both hosts together, lipid metabolism accounts for 50% of all induced DETs that were annotated with a known function (Supplementary Table S4).

Notably, *Desat1*, involved in unsaturated fatty acid biosynthesis, was among the most induced genes in the tobacco budworm (*Heliothis virescens*) hemocytes infected with *Helicoverpa zea single nucleopolyhedrovirus* [98]. Although it is associated with starvation-induced autophagy in *Drosophila* [99,100], many other integral components of the autophagy pathway known to be under transcriptional regulation [101] were not noticeably impacted in our study.

Whether host lipid synthesis genes are primarily involved in disease susceptibility or resistance is unclear. Distinguishing the specific involvement of these genes is challenging partly because of inadequate functional characterizations available for many of these genes in insect hosts. For example, in line with our results, previous studies have shown that *CG16904* is induced during parasitic infections [102], but its function is unknown. Similarly, *CYP4G1*, a cytochrome P450 gene involved in cuticular lipid synthesis and highly conserved in insects, has been identified as the most highly expressed among 85 of *CYP*450 genes in *Drosophila* [103]. Yet, the role of CYP4G1 during viral infections has not been elucidated, despite its direct functional association with the cuticle development. It is unclear how the host defenses lead to the up-regulation of these transcripts associated with lipid synthesis specifically during viral infections concurrently with the suppression of chitin-based processes and other structural components of the basement membrane. Based on the current study from intact infected hosts and supported by previous studies using cell cultures, it is imperative that the specific role of lipid synthesis in the complex host–pathogen interactions during AcMNPV infection are comprehensively investigated.

### 3.2. Hemocyte-Mediated Innate Immunity Is Suppressed during Systemic Infection

Hemolymph is the primary target tissue we used to deduce biological processes affected by the budded virus during systemic infections. Hemocytes in the hemolymph are known to elicit innate immune responses upon pathogen infections by phagocytosis, initiate agglutination via hemolectins and other associated proteins in hemostasis, initiate melanization, or inactivate pathogens via oxidants or other antimicrobial compounds [104]. In our study, we see prominent transcript signals that suggest a suppression of the hemocyte-mediated immune responses rather than transcriptional induction of those primary genes involved. This inference is supported by the coordinated suppression of *hemolectin* and *hemocytin* transcripts in infected *S. frugiperda* and *T. ni* hosts together with other transcripts such as the von Willebrand clotting factor (Figure 3, Figure 4b, Figure 6, and Supplementary Table S4).

Serine proteases and serine protease inhibitors play vital roles in hemocyte-driven phagocytosis, melanization, and antiviral immune responses in addition to their pleotropic functions in insect development [105,106]. The melanization reaction is tightly coupled to hemostasis reactions induced by hosts under pathogen infections as an integral part of the host immune response [46,47,48]. Yet, the detailed functional mechanisms of specific serine proteases in mounting defense responses against baculoviruses are poorly understood. In our study, a number of serine proteases and serine protease inhibitors were co-suppressed in both infected hosts (Figure 4b and Supplementary Table S4), implying a defense response compromised by the virus in infected hosts. It should be noted that both *S. frugiperda* and *T. ni* are known to be highly permissive hosts to AcMNPV infections [85,107]. Based on our results, the presence of the budded virus appears to strongly suppress the host immune responses initiated in the hemolymph.

### 3.3. Transcriptomic Signatures Indicative of Imbalances in Energy and Redox Homeostasis with Substantial Consequences to the Development of the Entire Organism during Disease Progression

Synergistic to maintaining membrane integrity via coordination of chitin and lipid metabolism, host cell survival depends on being able to maintain energy metabolism and redox homeostasis to minimize oxidative stress during infection and prevent further damage to membranes and DNA. Lavington et al. have demonstrated that a handful of enzymes in central energy metabolism can shift the flux balance and energy homeostasis [108]. Therefore, a failure to maintain homeostasis of these critical pathways suggests early signs of systemic progression of infection. We found multiple transcripts potentially coding for integral enzymes in energy metabolism and redox homeostasis suppressed in both host species by AcMNPV infection (Supplementary Table S4). These are often found to be regulated at the transcriptional levels. One such key enzyme in maintaining the redox pools and energy balance is the malic enzyme (coded by *Men* and *Men-b* genes) that catalyzes malate to pyruvate while reducing NADP to NADPH [108,109]. It has been estimated that 30% of the total cytosolic NADPH is produced by *Men* in *Drosophila* [110,111] and it is a critical enzyme in coupling energy metabolism to ROS levels under oxidative stress. Transcripts coding for the malic enzyme were suppressed with several other glycolytic transcripts in *T. ni*, while three out of the four significantly induced transcripts in *S. frugiperda* were transcripts associated with oxidative stress (Figure 4a and Figure 6, and Supplementary Table S4) [53,54,112].

The overall transcriptomic profiles in both infected hosts also suggest a compromised or reduced allocation of energy into critical larval development processes. We identified that a number of transcripts associated with development of wings, muscles, renal functions, and neurons in both infected hosts were significantly suppressed (Supplementary Table S4). Concurrently, we see a substantial fraction of ribosomal protein transcripts down-regulated in infected *T. ni* implying altered rates for protein translation and overall metabolism (Supplementary Table S4). This reduction is also observed in *S. frugiperda* but to a lesser magnitude (less than four-fold changes) (Supplementary Table S4). Taken together, these results suggest that critical cellular and metabolic processes seem to have been significantly affected, even if only 1% of the transcriptome in the infected hosts showed significant reduction in response to the AcMNPV infection. The impaired cellular and metabolic processes consequently may affect insect development.

### 3.4. Signaling Processes Associated with AcMNPV Infection

Several studies have discovered long-chain fatty acids used in novel pheromonal signaling pathways during larval stages that lead to aggregation of individuals unlinked from pheromonal signaling in reproduction [113,114,115,116,117]. Desaturase1 (Desat1) is a key enzyme associated with pleiotropic effects on pheromone production and perception [118,119]. Similarly, the lipid elongase gene, *bond* is known for its role in conspecific signaling [90]. *Desat1* and *bond* are co-induced in infected hosts (Figure 4a and Figure 6), while fatty acid biosynthesis and pheromone metabolism were among the enriched functions in response to AcMNPV infection in our study (Figure 6 and Supplementary Figure S4). The underlying genetic mechanisms of how pheromonal signaling pathways may have been exapted into a disease signaling pathway is unknown, but previous studies have confirmed the induction of these pathways in insects during viral infections [117,120,121]. The induction of a pheromonal pathway leading to conspecific aggregation during baculovirus infections could facilitate disease progression between individuals as non-infected larvae in close proximity to larvae that are undergoing liquefaction have a high risk in getting infected in the next disease cycle. Therefore, a pleiotropic gene such as *Desat1* is a likely candidate to be co-opted for behavioral traits evolved under an arms race between baculoviruses and their lepidopteran hosts. Alternatively, lipid synthesis genes could play a role in disease signaling systemically within the infected larvae by triggering ROS signaling [100,122]. The co-induction we observed for *GST* and other oxidative stress indicators (Figure 4a and Figure 6) in *S. frugiperda* may further support this idea of the involvement of ROS pathways in disease signaling.

### 3.5. The Role of AcMNPV Genes Found in the Host Hemolymph

The AcMNPV protein-coding genes regulate host cellular and physiological processes as well as the production of the two distinct types of enveloped virions: the occlusion-derived virions and the budded virions [123]. Our viral transcript quantification suggests that the budded virions are more abundant than occlusion-derived virions in infected hemolymph samples (Figure 5a and Supplementary Table S6), an observation also supported by previous studies [6]. The transcriptomic signature of the eukaryotic host genome is overrepresented compared to the viral genome expressed in our RNA samples that capture the host–pathogen interactions. Yet, the specific quantification of transcripts made feasible with RNA-seq data allows the detection of clear biological signals from the virus in host tissue (Figure 1 and Figure 5a, and Supplementary Table S6).

Baculoviruses arrest the molting of infected lepidopteran larvae [123]. This process is primarily governed by the ecdysteroid UDP-glucosyltransferase (EGT), a viral enzyme that inactivates the insect molting hormone, ecdysone [79]. *Ac15/Ac-egt* in the AcMNPV genome codes for EGT. In our study, *Ac15/Ac-egt* is among the most highly expressed viral transcripts found in the infected hemolymph of *S. frugiperda* and *T. ni* (Figure 5b and Figure 6, and Supplementary Table S6). Previous studies have reported higher EGT activities in the hemolymph compared to other tissue [124]. Hoover et al. demonstrated the role of EGT on the climbing behavior of gypsy moth larvae [125]. Subsequent studies have confirmed the role of EGT in influencing behavioral traits of other hosts including *T. ni* and *Spodoptera exigua* [126,127]. However, much of the genetic basis is unknown for these behavioral traits and at least in *Spodoptera* hosts, EGT alone is reported to be insufficient to elicit behavioral traits [126]. Therefore, the role of virus-induced behavioral effects on lepidopteran hosts remains a topic of interest at large.

### 3.6. Host Transcriptional Responses to the Budded Virus during the Systemic Infection Stage Differs from the Midgut Responses to the Occlusion-Derived Virus during the Primary Infection Stage

Shrestha et al. described the host transcriptomic landscape of the midgut during the primary infection phase of AcMNPV in *T. ni* 5th instar larvae primarily caused by the occlusion-derived virus [22]. The current study that focusses on the systemic infection stage predominantly caused by the budded virus in 4th instar larvae of two lepidopteran hosts including *T. ni* depicts a very different host transcriptomic landscape. The most consistently up-regulated transcripts (at least 16-fold) observed in the midgut in the study by Shrestha et al. included *REPAT* (*REsponse to PAThogens*), *Atlastin* (involved in ER and vesicle trafficking), *cyclic GMP-AMP synthase* (*cGAS*) genes that can bind to cytosolic viral DNA*, Ubiquitin-ligase-3 SIAH, a zinc finger CCHC,* a *peroxidase,* and a *chymotrypsin-like serine protease* [22]. None of these transcripts were found to be significantly expressed in response to the infection in the infected hemolymph in our study. An earlier study had shown increased *REPAT* in the midgut of baculovirus infections of *Spodoptera exigua* larvae [128] similar to the observations made by Shrestha et al. for *T. ni* [22]. These previous observations and the absence of significant changes to these transcripts in the hemolymph during systemic infections imply that these host transcripts may be specific to the infection phase or tissue. There is a clear transcriptomic signal given by multiple key apoptosis-related genes induced in the infected midgut of *T. ni*, as reported by Shrestha et al. [22]. While we do not observe the induction of the same transcripts in our study, several other apoptosis-related genes were suppressed in the infected hemolymph during systemic infections (Figure 6).

For certain time points post-infection in the midgut, Shrestha et al. reported up-regulation for several cuticle-related transcripts [22]. The transcriptomic signal associated with cuticle-proteins are likely stemming from tracheaoblasts in the hemolymph in our study, contrasting the transcripts reported by Shrestha et al. likely coding for cuticle-proteins affected in the peritrophic matrix lining the midgut during the occlusion-derived virus propagation [22]. The invasion of the budded virus into the tracheal epidermis is essential to the progression of the systemic infection as the host cannot shed these cells, unlike the gut epithelium infected by the occlusion-derived virus that can be shed as seen in semi permissive hosts [85]. This may explain why we observe exceedingly more transcripts potentially coding for cuticle, chitin, and associated membrane processes clearly suppressed as a result of successful disease progression than in infected midgut cells reported by Shrestha et al. [22].

The most notable consistently down-regulated genes (by at least 16-fold), during the occlusion-derived virus invasion of the midgut, mainly included orthologs of *flippase* and genes coding for a number of Cytochrome P450 enzymes*,* serine proteases, calcium binding protein P, and dehydroecdysone 3 alpha-reductase, as noted by Shrestha et al. [22]. None of these were significantly changed during the budded virus infection in either host in the current study. While there was hardly any direct overlap of down-regulated transcripts between the primary-midgut infection and the secondary-systemic infection, we see melanization as a suppressed pathway in both studies. Shrestha et al. described the down-regulation of *serine proteases* involved in the melanazation cascade similar to our observation for the suppression of multiple serine proteases thought to be involved in melanization and other defense responses (Figure 5b) [22]. Contrasting the overall observations made by Shrestha et al. during the midgut infection, the hemolymph of both hosts in our study during systemic infection appear to clearly induce transcripts associated with oxidative stress while suppressing those related to hemostatis, chitin metabolism, and tracheal development [22].

### 3.7. Key Host Genes Affected by the AcPNMV Infection Are Targets of Commercially Available Pesticides Used against Lepidopteran Pests

The commonly targeted host genes by the viral pathogen include *CHS1*, and transcripts associated with actin driven cellular functions as well as genes involved in insect hormone regulation. It is interesting to note that many of the chemical insecticides also use the same genes as primary targets to control lepidopteran pests. However, unlike chemical insecticides, baculoviruses continue to spread in the field post-host liquification.

Many insecticides target chitin biosynthesis as a more specific and safer alternative to generic insecticides such as pyrethroids and organophosphates. These chitin synthesis inhibitors largely include the benzoylphenylurea (BPU) group of insecticides, oxazolines, tetrazines, thiadiazines, thiazolidines, and triazines [129,130]. All chitin biosynthesis inhibitors act on chitin synthesis at various stages of the complex biochemical pathways, leading to the interruption of chitin production and cuticle development. The BPUs are shown to target *CHS1* to inhibit chitin metabolism early in the biosynthesis pathway [131]. Notably, *CHS1/kkv* is the main chitin synthase required for epicuticular stability, intact procuticle, maintenance of epidermal morphology, and sclerotization and pigmentation of the cuticle [132]. A number of genes associated with chitin synthesis and cuticle modifications (discussed earlier) are among the most highly suppressed transcripts in both hosts during systemic infection.

Pyridalyl is another commonly used potent insecticide against lepidopteran pests [131]. It has been used to control fall armyworm outbreaks in South Africa [133,134]. The molecular mechanism of pyridalyl generates excessive amounts of ROS, which eventually leads to severe oxidative stress and cell death in lepidopterans [135]. Among the handful of strongly induced genes during systemic infections, *GST* and other genes associated with oxidative stress are notable (Figure 4a and Figure 6). Further induction of these oxidative stress pathways disproportionately divert energy to oxidative stress responses that could expedite cell death and, in turn, host death. The current observation made in our study further supports the insecticidal potential of AcMNPV strains selected to induce host oxidative stress responses similar to observations made with pyridalyl activity.

Double stranded RNAs (dsRNAs) that mimic insect transcripts have emerged as a powerful tool for targeted pest control. For example, dsRNAs of actin transcripts used as foliar sprays have shown to be a promising insecticide for Colorado potato beetles that damage multiple Solanaceae crops [136]. In line with our findings made in the current study, baculoviruses are known to target actin-mediated cellular processes [68]. Actin is present in all cells and customization to target-specific lepidopteran actins or a regulatory gene of actin-mediated processes is equally achievable with baculoviruses. Transcript-level inhibition of the juvenile hormone biosynthesis or alterations to its regulation is another target attempted in recombinant baculoviruses developed as potential biopesticides [137,138]. Host genes associated with juvenile hormone regulation were noticeable among suppressed transcripts in infected *T. ni* in our study similar to previous observations made when wild type AcMNPV strains were used [139]. Importantly, baculoviruses are more effective as delivery agents in controlling host genes than the passive delivery methods available for dsRNA-based insecticides [140]. The use of recombinant baculovirus strains to control pests has been proposed for decades and has recently gained more attention as sustainable biopesticides [141,142,143].

## 4. Material and Methods 

### 4.1. Insect and Virus Source Material

Given the natural progression of an epizootic in the field and the need to collect a considerable amount of hemolymph for the transcriptome analysis, we used 4th larvae in the experiments outlined below. *S. frugiperda* and *T. ni* were obtained as eggs from Benzon Research Inc. (Carlisle, PA, USA). Once the eggs hatched, we reared them in individual one-ounce cups on an artificial diet (Southland Products Inc., Lake Village, AR, USA) at 28.9 °C and a 16 hour-light and 8 hour-dark cycle until they reached the 4th instar. Wild-type AcMNPV strain E2, which was used in this study, was field collected. To amplify the virus for the experiment, the virus was passed through *Chrysodeixis includens*, the soybean looper.

### 4.2. Determination of LD95 of AcMNPV for S. frugiperda and T. ni

We used a standard dose-response protocol and Bayesian analysis to quantify the lethal dose at which 95% of the larvae would be expected to succumb to viral infection or the LD95. For the experiment, 30 recently molted 4th larvae, which were starved for 24 h, were fed a known amount of virus on a small diet cube. The virus was suspended in a 3 μl droplet of deionized water. One set of larvae was used as a control and consumed a diet cube that had been only inoculated with 3 μls of deionized water. None of the controls became infected. Only larvae that consumed the entire diet cube were used in the experiment to ensure that the larvae received a full dose of the virus. Viral doses varied depending upon the species (Figure 1). After consuming the diet cube, larvae were placed in one-ounce cups and reared until pupation or death. Death resulting from AcMNPV infection was confirmed either by host liquefaction in the diet cup or by examining the hemolymph under a light microscope [8]. For *T. ni*, the experiment was conducted twice, since the first set of experiments used doses that were too high resulting in almost 100% mortality and, thus, making it difficult to estimate the LD95. The second set of experiments used much lower doses. We combined the data from the two experiments for the *T. ni* dose-response analysis.

To analyze the data, we used a Bayesian framework with vague priors to fit a logistic regression model [144] for each species. The associated slope and intercept of the fitted model was used to calculate the LD95. All analyses were conducted in R [145] using the JAGS [146] and the R2JAGS packages [147]. For each analysis, three chains were run from different starting points. The first 10,000 draws from the Bayesian Markov chain Monte Carlo (MCMC) were removed to account for transient dynamics at the start of the chain. The remaining 90,000 draws were retained to estimate the parameters of the logistic regression. All non-discarded draws were retained to ensure precise parameter estimates [148]. After a visual inspection of the chains for convergence, multiple tests were used to ensure that the chains had converged including the Gelman-Rubin and the Hiedelberg-Welch tests [149]. The chains for each analysis were combined to form a posterior distribution. Additionally, we conducted a posterior predictive check to test whether the predicted model fit the data collected [150]. As part of the posterior predictive check, Bayesian *p*-values were calculated. Values near 0.50 indicate that the model fits the data reasonably well [151]. The Bayesian logistic regression for both species passed each of the individual tests outlined above.

### 4.3. Insect Treatment with AcMNPV Virus

Using the LD95 calculated from the dose-response experiments, 4th instar larvae from both species were fed the appropriated dose of virus (*S. frugiperda*, 10^4.5^ occlusion bodies *T. ni*, 10^3^ occlusion bodies) in a diet cube using the same method as the dose-response experiment. Control larvae were fed a diet cube inoculated with deionized water. After 30 hours, 30 individuals per sample were used to extract the hemolymph.

### 4.4. Extraction of Hemolymph and Preparation of RNA-Seq Libraries

Prior to hemolymph extraction, each individual was chilled to ease the extraction process. A pre-chilled microcentrifuge tube was filled with a 25 μL solution containing 10 units of RNAseOut (Invitrogen, Carlsbad, CA, USA) in a 0.1 % PTU dissolved in a PBS solution. The rear proleg of the 4th instar larva was then cut with micro scissors. We collected hemolymph from the wound and pipetted the hemolymph into a pre-chilled Eppendorf tube (USA Scientific, Inc., Ocala, FL, USA). The solution was then vortexed, immediately placed in a dewar filled with liquid nitrogen, and stored at −80 °C until needed. Given that the previous publication has reported that the hemolymph contains occlusion bodies in a relatively short time post infection [152], we extracted the hemolymph and used it for our transcriptome analysis. The hemolymph includes hemocytes as the major blood cell type. Other cell types, including tracheoblasts, midgut epithelial cells, fat bodies, nerve, muscle, and other epithelial cells lining internal organs, are immersed or in contact with the hemolymph and can release proteins, metabolites, and transcripts into the cell-free fraction of the hemolymph. We discarded any hemolymph sample that had visible tissue contamination when drawing it from larvae but expect to see transcripts from the above sources in the RNAseq analyses of the hemolymph.

Total RNA was isolated from hemolymph samples using RNeasy Mini Kit (Qiagen, Hilden, Germany). On-column DNase digestion was carried out with the RNase-free DNase Kit (Qiagen, Hilden, Germany), followed by a further purification step using RNeasy Mini Spin Columns (Qiagen, Hilden, Germany). The quantity, quality, and integrity of the total RNA was sequentially assessed using the A260/A280 values reported with a Nanodrop spectrophotometer (Thermo Scientific, Wilmington, DE), agarose gel (Agarose unlimited^TM^, Gainsville, FL, USA) electrophoresis, and a BioAnalyzer (Agilent Technologies, Inc., Santa Clara, CA, USA).

RNA-seq library preparation and sequencing were done at the University of Illinois at Urbana-Champaign Roy J. Carver Biotechnology Center. Ribosomal RNA (rRNA) depletion was performed on the RNA samples using the RiboZero kit (Illumina, San Diego, CA, USA) following the manufacturer’s instructions. Capturing polyA-enriched RNA from total RNA is a more customary approach for eukaryotic RNA-seq experiments. However, we decided to use rRNA depleted samples because we planned to identify both insect and viral transcripts which may not always contain 3′ polyA sequences. The rRNA-depleted samples were used for TruSeq Stranded RNA Sample Prep kit to produce 5′ to 3′ strand-specific cDNA libraries (Illumina, San Diego, CA, USA). A TruSeq SBS sequencing kit version 3 (Illumina, San Diego, CA, USA) was used following the manufacturer’s instructions to generate the sequencing libraries. All libraries were pooled, barcoded, and multiplexed on two lanes of an Illumina HiSeq2000 platform (Illumina) to run for 101 cycles. Randomly selected reads of 100 nucleotide lengths from each library were processed and demultiplexed with Casava 1.8.2, which generated over 370 million reads with quality scores over 30.

### 4.5. Sequencing, Assembly, and Annotation of the Reference Transcriptome

To allow accurate identification of host transcripts from two species, we needed to create two reference transcriptomes for the hemolymph of 4th instar caterpillars. RNA-seq reads were processed to generate a reference transcriptome assembly and annotation following a custom pipeline published previously [153]. Briefly, raw Illumina reads were subjected to quality checks using FastQC and de novo assembled using Trinity v2.2.0 [154] using default parameters. Contigs with low read support, contaminants, and artifacts were removed as described in Oh et al. [155]. We further clustered contigs showing >95% sequence identity over >70% of total contig length of the shorter contig in each pairwise alignment, using CD-HIT-EST v4.6 [156] to minimize redundancy. For each cluster, the transcript with the longest open reading frame (ORF), predicted by Transdecoder v2.0.1 (https://transdecoder.github.io/), was selected as a representative transcript model in the final protein-coding reference transcriptome. The completeness of each reference transcriptome assembly was evaluated using Benchmarking Universal Single-Copy Orthologs (BUSCO) database v2.1 [28] with the metazoan dataset (metazoa_odb9) and default settings. BUSCO looks for the presence of the conserved single copy orthologs in the selected lineage, which we chose as metazoans, which included 65 species. A series of sequential BLAST searches found the best possible annotation for both coding and non-coding transcript sequences, using the NCBI *Drosophila* mRNA database, NCBI-insects-reference RNA (refseq_rna), and NCBI-non redundant (nr) databases for all eukaryotic proteins and RNA, with a maximum e-value cutoff of 10^−5^.

An ideal transcriptome is expected to consist of all expressed genes in a given condition. This would include both coding and non-coding transcripts. However, the non-coding transcript pool is highly incomplete even for the premier model species. Therefore, it would be impractical to assign reasonable functional annotations for contigs that may represent true non-coding transcripts in our study. Additionally, without any canonical structural features to use in assessing the completeness of non-coding transcripts, those transcripts could also contain a highly fragmented fraction of the assembly. Therefore, we divided our assembled transcriptome into coding and non-coding reference transcriptomes and only used the protein-coding transcriptome for our current analyses. Despite the lack of resources to fully annotate putative non-coding transcripts, this pool of non-coding transcripts likely represents a genetic component that has potential to be useful as a collective resource from diverse species as more high throughput data-driven projects are conducted. Therefore, we include Table 1 and Supplementary Figure S1, where we report a total of 101,169 and 147,772 processed non-coding transcripts, with a mean length of 495 and 549nt, for *S. frugiperda* and *T. ni*, respectively, as an additional molecular resource included in our data deposit to NCBI BioProject PRJNA664633.

The protein-coding reference transcriptome was used for the downstream RNA-seq analysis. Each sequence used as a proxy to represent gene/transcript models in our study when assessing biological processes is designated by its gene name, followed by the shortened form of the gene name (if available), the sequence ID given by our annotation process, and the FlyBase or NCBI accession number used for its annotation as shown in Supplementary Table S4. The highly abundant or regulated genes discussed in our study are listed in Supplementary Table S7 with references that describe their molecular function.

### 4.6. RNA-Seq Analysis

The goal of our experiment was to search for shared disease responses inferred from the two host species affected by AcMNPV infection using three sets of biologically independent RNA-seq datasets. Two datasets were from *S. frugiperda* and one set was from *T. ni.* The RNA-seq reads were aligned to the relevant reference transcriptome using bowtie [157] with a seed alignment length per read set to 50 nt. Reads unambiguously mapped to each gene model were counted using a custom python script to generate read-count values as a proxy for gene expression. We used NOISeq [158] with a q-value cutoff of ≥0.95 to identify transcripts differently expressed between control and AcMNPV-infected samples in both insect species. Gene ontology (GO) terms enriched among differently expressed transcripts (DETs) were detected using BiNGO [159] at a false discovery rate (FDR) adjusted *p*-value < 0.05 [159]. We used the entire reference protein-coding transcriptomes as custom backgrounds to test for functionally enriched clusters when inferring the shared biological processes identified from each host species. GO annotation of reference protein-coding transcriptomes for the two insect species was based on sequence similarity compared to *Drosophila melanogaster* gene models that have assigned GO terms. We used GOMCL [41] to identify the non-redundant functional clusters from the primary set of enriched functions generated using BINGO for each species.

To assess the transcripts originating from the viral genome, particularly in the infected samples, RNA-seq reads were mapped to the AcMNPV reference genome gene models [4] using bowtie [157]. The read counts mapped to the viral genome were normalized by converting to RPKM values (Reads Per Kilobase Million) for each viral gene expressed in the insect transcriptomes. Total read counts were calculated by adding the reads mapped to the viral genome and insect gene models for control and AcMNPV infected samples as used in a previous study [160].

## 5. Conclusions

We identified an extensive overlap between biological processes that were represented by differently expressed transcript in 4th instar larvae of two hosts, *S. frugiperda* and *T. ni*, in response to the systemic infection of AcMNPV as well as convergence in the ranked abundance of viral genes expressed in the two hosts. The overall host transcriptomic signals suggested chitin-associated processes and membrane integrity were compromised together with immune responses in infected hosts. Our results suggest that oxidative stress indicators, moderately induced by the viral infection, may play a role in systemic disease signaling with the induction of selected classes of fatty acids (Figure 6). The entire core viral genome was detected during the systemic infection phase in both hosts, with a bias towards transcripts primarily associated with the budded form of the virus. The host–virus interactions deduced from co-expressed host and viral transcripts indicate an overall transcriptomic landscape overwhelmed by viral counter defenses that facilitate disease progression. The specific transcripts and the convergent biological processes, highlighted in our study as highly affected during infections, identify key genes and pathways as potential molecular targets that may contribute to designing recombinant AcMNPV strains as molecular tools in sustainable pest management.

## Figures and Tables

**Table 1:** Summary of de novo assembled reference transcriptomes of *S. frugiperda* and *T. ni.*

Transcriptome Assembly Features	*S. frugiperda*	*T. ni*
	Coding ^a^ (Non-Coding ^b^)	Coding ^a^ (Non-Coding ^b^)
Total assembled transcripts	17,908 (101,169)	19,472 (147,934)
Percent GC	42.68 (35.28)	41.16 (35.24)
Contig N50 (nt)	2279 (532)	2955 (656)
Average contig length (nt)	1458 (495)	1773 (549)
Smallest contig length (nt)	297 (224)	297 (201)
Longest contig length (nt)	29,765 (14,343)	30,823 (23,482)
Number of ORFs	28,433 (-)	31,292 (-)
Average ORF length (nt)	920.28 (-)	841.86 (-)
Smallest ORF length (nt)	297 (-)	297 (-)
Longest ORF length (nt)	27,558 (-)	27,408 (-)
Mapped sequenced read % to the reference assembly	76	84
Detected complete BUSCOs (%) ^c^ (Arthropoda reference)	80.6	82.1

^a^ represents transcript models with a predicted open reading frame (ORF); ^b^ represents transcript models without a predicted ORF; ^c^ BUSCO (Benchmarking Universal Single-Copy Orthologs) v1.22 [28].

## Data Availability

The Illumina RNA-Seq reads from this article have been deposited to the NCBI sequence read archive (SRA) database, the assembled transcriptome is available in transcriptome shotgun assembly (TSA) database, and the expression profile is available at gene expression omnibus (GEO) under the accession number for NCBI BioProject ID: PRJNA664633.

## Supplementary Materials

The supplementary materials are available online at https://www.mdpi.com/article/10.3390/ijms22073568/s1. Figure S1. Assembled contig length frequency distribution for *S. frugiperda* and *T. ni* reference transcriptomes. [a] Coding contigs and [b] non-coding contigs. Comparison of average CDS length [c] and number of protein coding transcripts [d] between *S. frugiperda* and *T. ni* compared to *B. mori*, *H. armigera, S. litura*, and the *T. ni* genome. Figure S2. Quality assessments of *S. frugiperda* and *T. ni* reference transcriptomes. [a] proportions of different ORF types and [b] assembly completeness of the references created in this study compared to the previously published *S. frugiperda* draft genome and transcriptomes (Kakumani et al. 2014 and Legeai et al. 2014) assessed using BUSCO. Figure S3. Annotation summary of the *S. frugiperda* and *T. ni* transcriptome assembly for coding transcripts. Functional annotation of reference transcriptome was performed using sequential BLAST with an e-value cutoff 10^−5^ searched within the *Drosophila* mRNA database, insect reference RNA (refseq_rna) database, and non-redundant (nr) database. The annotations of reference transcriptome for both species are provided in the Supplementary Table S3. Figure S4. Principal component analysis (PCA) of ortholog gene pairs between *S. frugiperda* and *T. ni.* Figure S5. Clustered enriched functional processes among induced *T. ni* transcripts upon AcMNPV infection. The full list of enriched GO terms and GOMCL cluster output are included in the Supplementary Table S5. Figure S6: Amino acid sequence alignment of *Ac-IE-1/Ac147*, *Ac-IE-0,* and *Ac-IE-01. Ac-IE-1* shows high similarity to *Ac-IE-01* except for the 54 amino acids in the amino terminus end of *Ac-IE-01*. The sequence alignment was performed with CLUSTAL O (1.2.4). Previously known *Ac-IE-01* is annotated as *Ac-IE-0* and previously known *Ac-IE-0* is annotated as *Ac-IE-01* in the AcMNPV genome clone C6, gene bank accession number: L22858 (Ayres et al., 1994) and AcMNPV E2 genome: gene bank accession number: KM667940 (Maghodia et al., 2014). Table S1. RNAseq data generated for each sample. Table S2. [a] Summary of short reads mapped to *S. frugiperda* and *T. ni* reference transcriptomes and [b] percentage of short reads mapped to the AcMNPV viral genome (Maghodia et al. 2014) for control and AcMNPV treated samples. Table S3. Annotation of transcript models with predicted ORFs for *S. frugiperda* and *T. ni* transcriptome assembly. Table S4. List of DETs for *S. frugiperda* and *T. ni* in response to AcMNPV infection. Table S5. Gene ontology enrichment analysis for DETs for *S. frugiperda* and *T. ni.* Table S6. Reads associated with AcMNPV genes mapped to *S. frugiperda* and *T. ni* control and infected samples. Table S7. References to molecular functions associated with genes highlighted in our analysis.
