# Supplementary material for: A Tale of Two Transcriptomic Responses in Agricultural Pests via Host Defenses and Viral Replication
Source: Int J Mol Sci. 2021 Mar 30;22(7):3568. doi: 10.3390/ijms22073568 (PMC8037200; doi:10.3390/ijms22073568)
Supplement: Supplementary file 1 [file ijms-22-03568-s001.zip › ijms-1156750-supplementary/ijms-1156750-supplementary.docx]

**Supplementary Materials:**

**
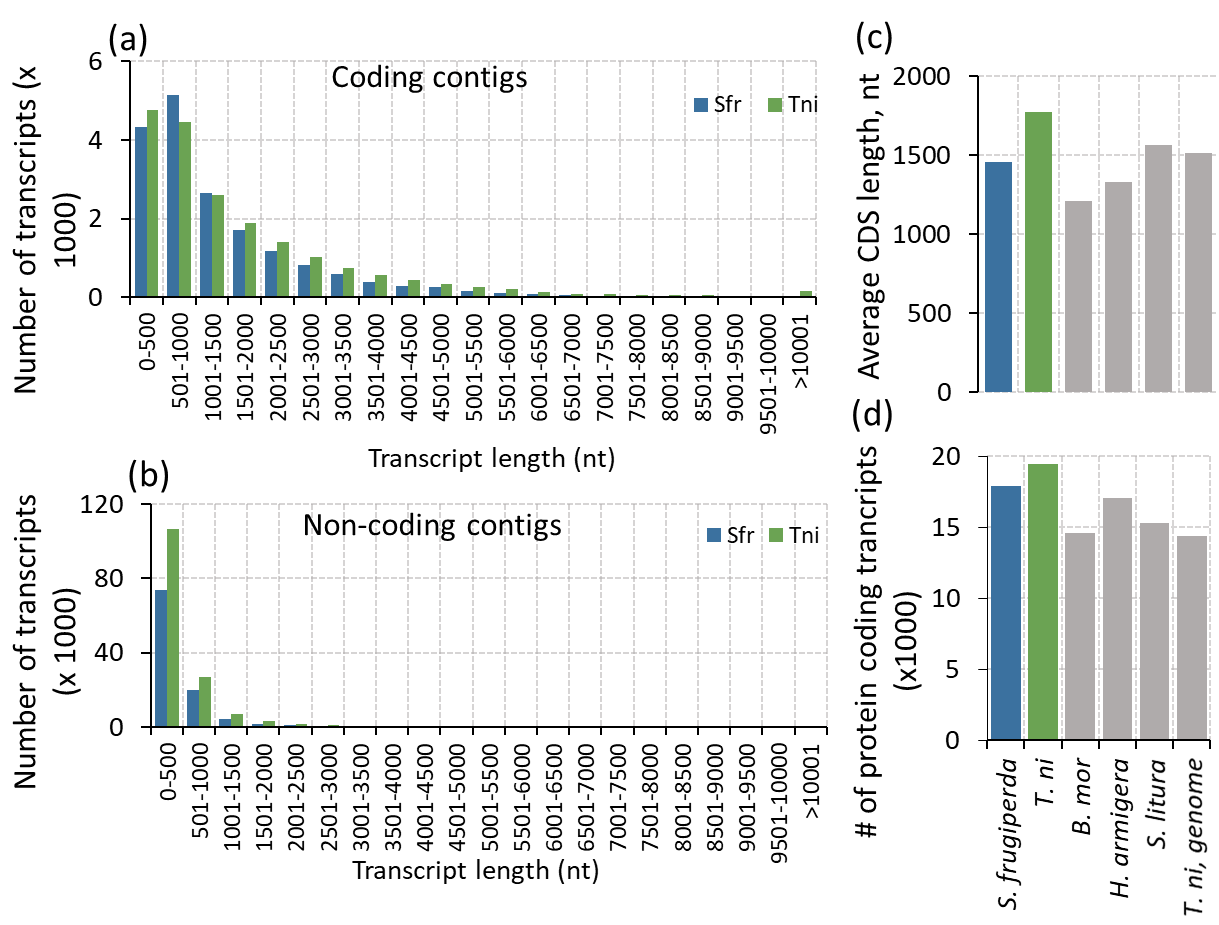
**

**Supplementary** **Figure S1.** Assembled contig length frequency distribution for *S. frugiperda* and *T. ni* reference transcriptomes. [a] Coding contigs and [b] non-coding contigs. Comparison of average CDS length [c] and number of protein coding transcripts [d] between *S. frugiperda* and *T. ni* compared to *B. mori*, *H. armigera, S. litura*, and the *T. ni* genome.

**
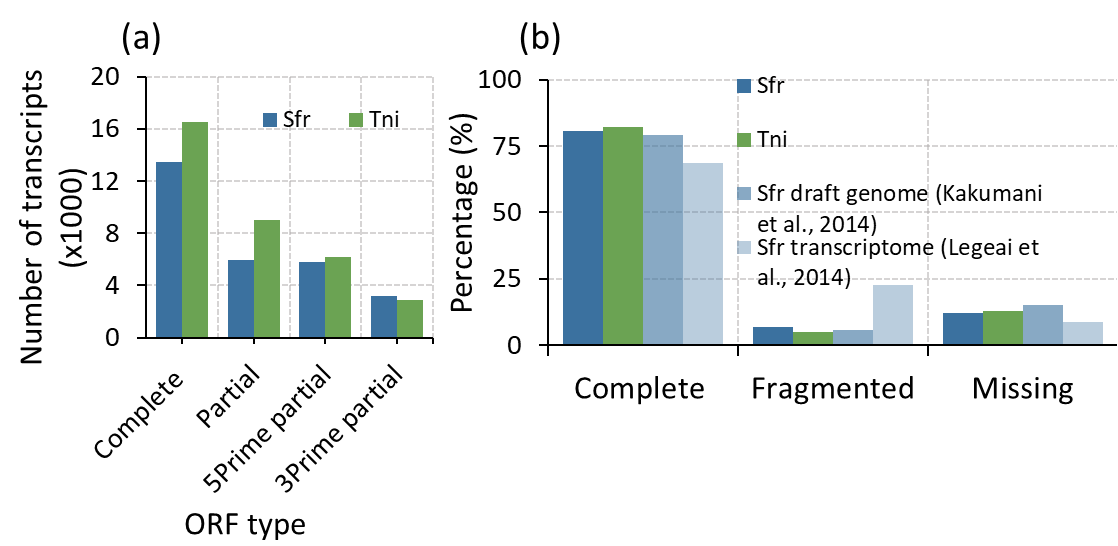
**

**Supplementary** **Figure S2.** Quality assessments of *S. frugiperda* and *T. ni* reference transcriptomes. [a] proportions of different ORF types and [b] assembly completeness of the references created in this study compared to the previously published *S. frugiperda* draft genome and transcriptomes (Kakumani et al. 2014 and Legeai et al. 2014) assessed using BUSCO.


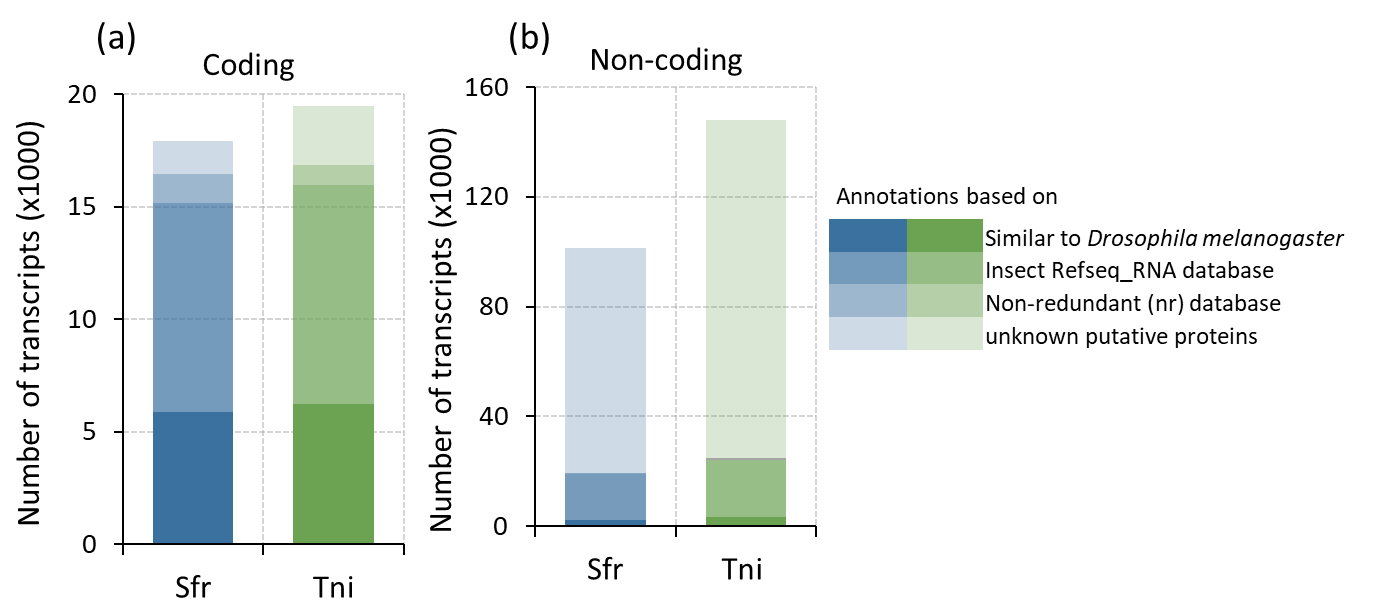


**Supplementary** **Figure S3.** Annotation summary of the *S. frugiperda* and *T. ni* transcriptome assembly for coding transcripts. Functional annotation of reference transcriptome was performed using sequential BLAST with an e-value cutoff 10^-5^ searched within the *Drosophila* mRNA database, insect reference RNA (refseq_rna) database, and non-redundant (nr) database. The annotations of reference transcriptome for both species are provided in the Supplementary Table 3.

**
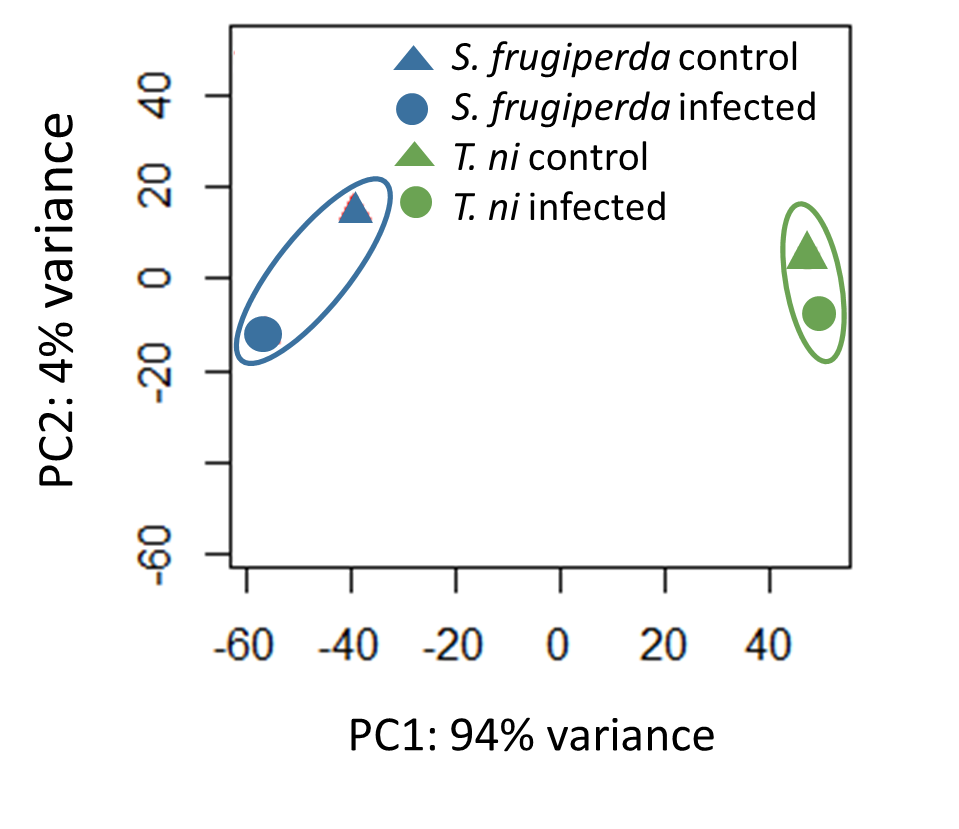
**

**Supplementary** **Figure S4.** Principal component analysis (PCA) of ortholog gene pairs between *S. frugiperda* and *T. ni.*

**
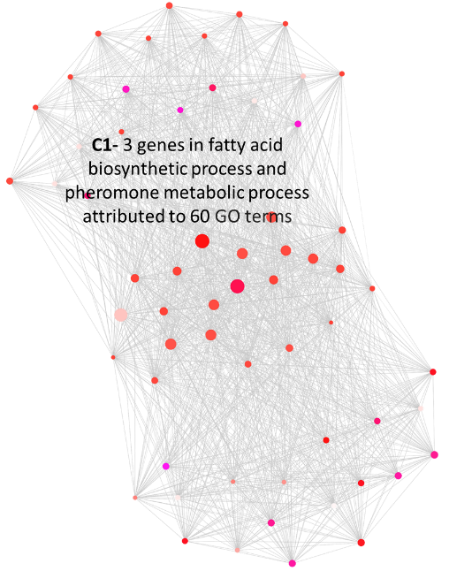
**

**Supplementary** **Figure S5.** Clustered enriched functional processes among induced *T. ni* transcripts upon AcMNPV infection. The full list of enriched GO terms and GOMCL cluster output are included in the Supplementary Table 5.


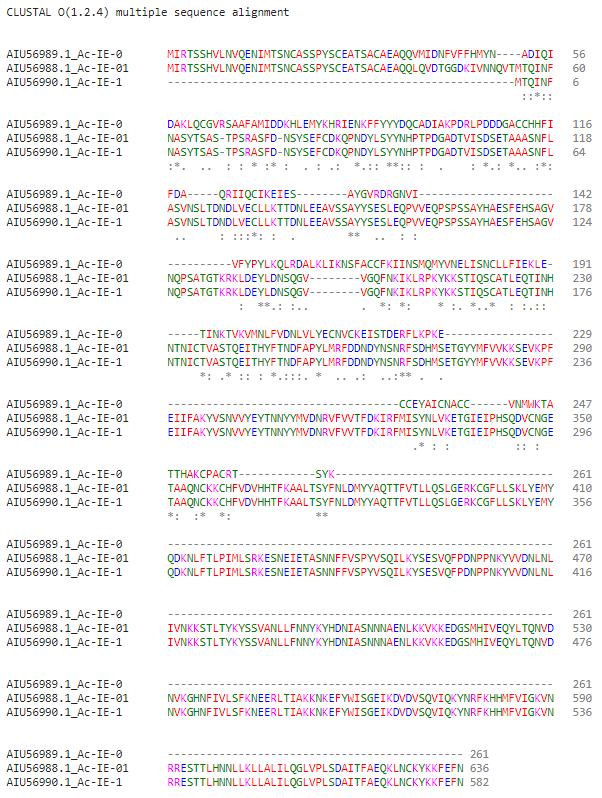


**Supplementary** **Figure S6:** Amino acid sequence alignment of *Ac-IE-1/Ac147, Ac-IE-0,* and *Ac-IE-01. Ac-IE-1* shows high similarity to *Ac-IE-01* except for the 54 amino acids in the amino terminus end of *Ac-IE-01*. The sequence alignment was performed with CLUSTAL O (1.2.4). Previously known *Ac-IE-01* is annotated as *Ac-IE-0* and previously known *Ac-IE-0* is annotated as *Ac-IE-01* in the AcMNPV genome clone C6, gene bank accession number: L22858 (Ayres et al., 1994) and AcMNPV E2 genome: gene bank accession number: KM667940 (Maghodia et al., 2014)**.**

**Supplementary** **Table S1.** RNAseq data generated for each sample.

**
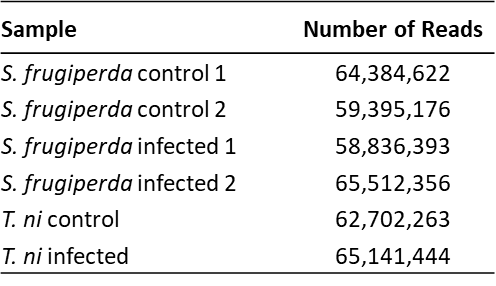
**

**Supplementary** **Table S2.** [a] Summary of short reads mapped to *S. frugiperda* and *T. ni* reference transcriptomes and [b] percentage of short reads mapped to the AcMNPV viral genome (Maghodia et al. 2014) for control and AcMNPV treated samples.

**
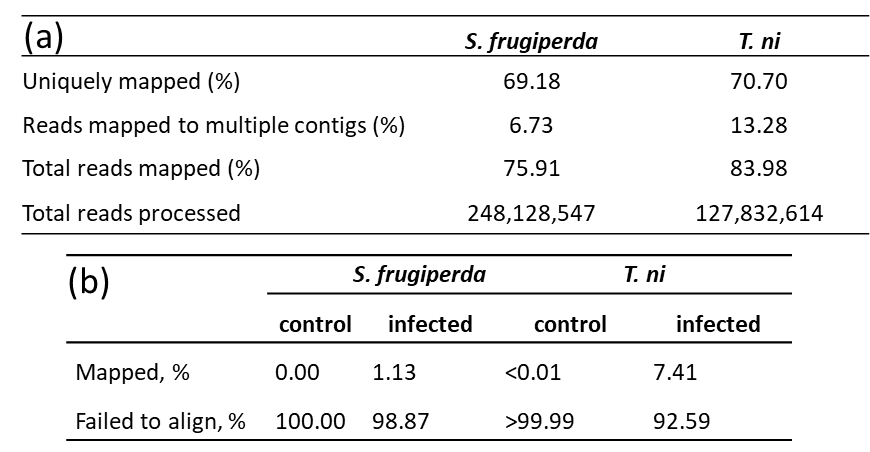
**

**Supplementary Table S3.** Annotation of transcript models with predicted ORFs for *S. frugiperda* and *T. ni* transcriptome assembly.

**Supplementary Table S4.** List of DETs for *S. frugiperda* and *T. ni* in response to AcMNPV infection.

**Supplementary Table S5.** Gene ontology enrichment analysis for DETs for *S. frugiperda* and *T. ni.*

**Supplementary Table S6**. Reads associated with AcMNPV genes mapped to *S. frugiperda* and *T. ni* control and infected samples.

**Supplementary Table S7.** References to molecular functions associated with genes highlighted in our analysis.
